# PD26 Cost Effectiveness Of Endoscopic Retrograde Cholangiopancreatography (ERCP) With Cholangioscopy For Difficult Biliary Stones After Failed ERCP: Brazilian Health Sector Perspective

**DOI:** 10.1017/S026646232510161X

**Published:** 2025-12-29

**Authors:** Henrique Tortele, Maiara Antonio, Anna Pereira, Murilo Contó

## Abstract

**Introduction:**

Patients with difficult biliary stones often require repeated conventional ERCP procedures or others surgical interventions, leading to complications and unnecessary expenses for the healthcare system. ERCP with cholangioscopy allows direct visualization and use of laser or electrohydraulic lithotripsy, which are not possible without cholangioscopy. This study aimed to evaluate the cost effectiveness of ERCP with cholangioscopy for treating difficult biliary stones.

**Methods:**

A decision tree model was developed to estimate the cost effectiveness of ERCP with cholangioscopy for direct visualization. Patients started the model with difficult biliary stones after an initial failed ERCP procedure. In the base scenario, patients underwent a second conventional ERCP procedure. In the proposed scenario, cholangioscopy was used in the same procedure immediately after the first failed ERCP. In the case of technical success with complete removal of the stones, patients exited the model, otherwise surgery was performed. Estimates of success rate, resources used, and costs were obtained from the literature or expert opinion and estimated from the supplementary health sector’s perspective.

**Results:**

ERCP with cholangioscopy used immediately after a failed conventional ERCP in the same procedure was cost-effective and dominant alternative. It increased success rates for removing difficult biliary stones and reduced resource use and complication rates, benefiting the Brazilian supplementary health sector. This approach offered significant advantages over repeated conventional ERCP procedures or surgical interventions.

**Conclusions:**

Reducing the number of repeated attempts at endoscopic extraction of complex gallstones prevented several adverse patient outcomes and reduced healthcare resource utilization, medical costs, safety implications, and delays to more effective treatment.

